# Rheological and Physical Properties of Mucilage Hydrogels from Cladodes of *Opuntia ficus-indica*: Comparative Study with Pectin

**DOI:** 10.3390/gels11070556

**Published:** 2025-07-19

**Authors:** Federica Torregrossa, Matteo Pollon, Giorgia Liguori, Francesco Gargano, Donatella Albanese, Francesca Malvano, Luciano Cinquanta

**Affiliations:** 1 Department of Agricultural, Food and Forestry Sciences, University of Palermo, Viale delle Scienze 4, 90128 Palermo, PA, Italy; federica.torregrossa01@unipa.it (F.T.); matteo.pollon@unipa.it (M.P.);; 2 Department of Industrial Engineering, Università di Salerno, Via Ponte Don Melillo, 84084 Fisciano, SA, Italyfmalvano@unisa.it (F.M.)

**Keywords:** mucilage, pectin, *Opuntia*, ·rheological properties, ionic strength, viscosity

## Abstract

The physical and rheological properties of mucilage hydrogels derived from the cladodes of *Opuntia ficus-indica* (L. Mill) were compared with those of commercial pectin for potential applications in the food industry. All hydrogels—formulated by incorporating sucrose and either calcium chloride or calcium carbonate to promote favorable gel network formation—exhibited pseudoplastic (shear-thinning) behavior. The flow characteristics of the hydrogels prepared with mucilage or pectin conformed to the Casson fluid model. Moreover, all samples consistently displayed loss modulus (G″) values exceeding their corresponding storage modulus (G′) values, indicating a dominant viscous behavior over elastic properties. The ζ-potential of all samples was negative across the pH range studied. Mucilage-based samples exhibited lower ionizability per unit mass and reduced phase stability compared to those containing pectin. Principal component analysis (PCA) revealed that mucilage hydrogels exhibited multivariate profiles similar to pectin hydrogels containing calcium carbonate, though the latter demonstrated greater polydispersity than standard pectic gels. Infrared spectroscopy further highlighted distinct spectral differences between pectins and mucilages, offering valuable insights into their respective functional characteristics. Collectively, these findings underscore the potential of *Opuntia ficus-indica* mucilages as viable additives in food formulations.

## 1. Introduction

The use of polysaccharides in foods has increased significantly, stimulating the search for new hydrocolloids with favorable functional properties or economic advantages over existing options. Among these, pectin is widely used as a gelling, thickening, or stabilizing agent in baked goods, confectionery, and dairy products due to its ability to trap water, form hydrogels, and create an elastic network [1]. Pectin is a naturally occurring heteropolysaccharide of dietary interest of which its multifunctionality is due to the nature of its molecule, which contains both polar and nonpolar regions, allowing it to be incorporated into various food systems. Among its many functional properties, pectin’s ability to form gels is particularly important and is attributed to hydrogen bonding between hydroxyl groups of D-galacturonic acid chains [2]. As such, it can be considered a fat replacer, a dietary fiber, and a prebiotic [3].

The gelling capability of pectin is primarily due to hydrogen bonding between the hydroxyl groups of D-galacturonic acid chains [2]. High methoxyl pectins (HMPs), which are commonly used in the food industry, exhibit desirable gelling properties in acidic and high-sugar environments. Their rheological stability, thermo-reversibility, application versatility, and cost-effectiveness make them suitable for a wide range of food products [4]. Sucrose and calcium ions (Ca^2+^) act synergistically to enhance the structural strength of HMP-based gels. Gel formation is particularly effective at low Ca^2+^ concentrations (0–0.35% *w*/*w*), while high sucrose concentrations reduce water activity and promote hydrophobic interactions between methoxyl groups [5].

Current research is increasingly focused on developing new hydrocolloids—long-chain polymers derived from agricultural by-products—capable of forming functional gels [6]. Mucilage, which plays a crucial role under water stress conditions [7], is a complex carbohydrate composed of varying amounts of L-arabinose, D-galactose, L-rhamnose, D-xylose, and galacturonic acid. Accordingly, it can be classified as a native hydrocolloid with diverse functional and technological properties, making it valuable for applications in the food industry as a thickening agent, emulsifier, and stabilizer [8]. The applications of *Opuntia ficus-indica* (OFI) mucilage have been reported in the formulation of mayonnaise, baked goods, and dairy beverages. The rheological properties of OFI mucilage also render it a promising candidate for the development of natural edible coatings with high nutraceutical value, potentially useful for food and fruit preservation [9].

Thus, mucilage extracted from members of the Cactaceae family represents a potential source of food additives [10], capable of enhancing the textural properties and functionality of foods, primarily through their physical characteristics such as gelling ability. Generally, gel formation depends on the molecular weight and spatial arrangement of the biopolymer; in the case of pectin, factors such as the pH, salt presence (particularly Ca^2+^), and degree of esterification further influence gelation [11]. Given the potential use of OFI mucilage as a food ingredient, as a fat replacer in low-fat products (i.e., low-fat yogurt, mayonnaise, dressings, processed cheese), or as a natural thickening and gelling agent (i.e., sauces, gravies, soups, jams, jellies), the present study aims to characterize the physicochemical and rheological properties of mucilage extracted from *O. ficus-indica* cladodes and to compare them with those of pectin commonly utilized in the food industry. This is particularly relevant as literature data on dried mucilage from OFI cladodes remain limited. Accordingly, the gel-forming capacity and properties of these polysaccharides were evaluated under various conditions and in the presence of different salts, with comparisons made to commercial pectin. The effects of the concentration, pH, ζ-potential, and particle size distribution on flow behavior and viscoelastic properties were also investigated.

## 2. Results and Discussion

### 2.1. Flow Curve Analysis

**Figure 1:**
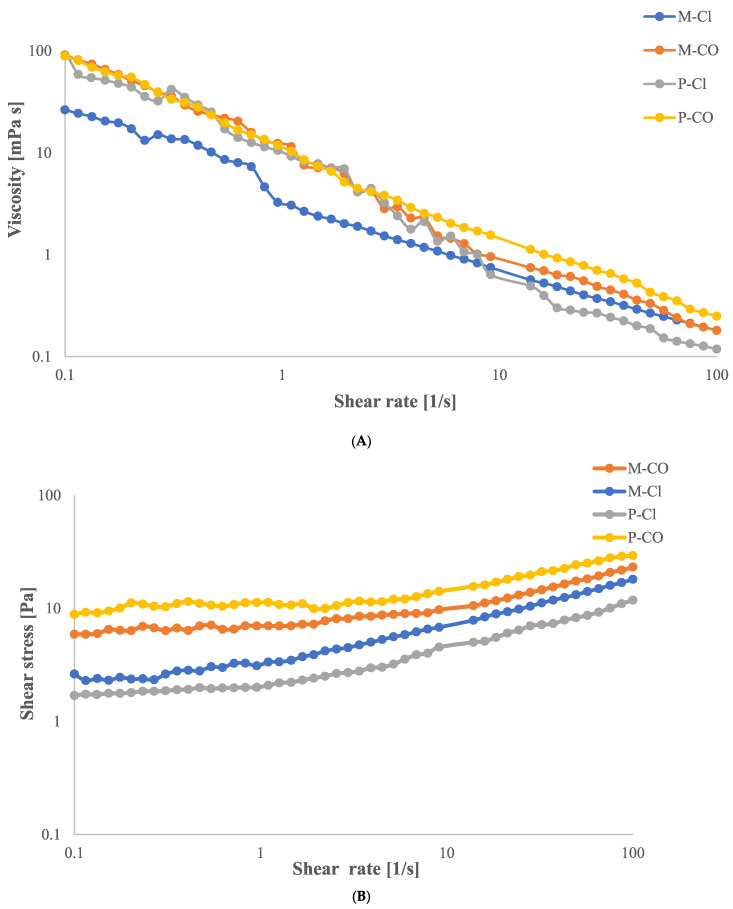
Viscosity curves (**A**) and flow curves (**B**) for hydrogels: mucilage added to CaCl_2_ (M-Cl); mucilage added to CaCO_3_ (M-CO); pectin added to CaCl_2_ (P-Cl); pectin added to CaCO_3_ (P-CO).

As shown in Figure 1A, all hydrogel samples exhibited non-Newtonian behavior, as evidenced by the change in apparent viscosity with an increasing shear rate. In particular, the flow curves indicate a pseudoplastic (shear-thinning) behavior, in which the apparent viscosity decreases as the shear rate increases. This phenomenon can be attributed to the progressive alignment of polymer chains in the direction of flow at higher shear rates. Such alignment leads to a reduction in intermolecular interactions between adjacent chains, thus facilitating chain mobility and resulting in a lower resistance to flow. This behavior is typical of weakly structured or cross-linked systems, where the applied mechanical stress causes partial disruption of the gel network [12].

Moreover, as shown in Figure 1B, all hydrogels display an initial critical shear stress (yield stress), below which no flow occurs. Only when this threshold is exceeded does the applied stress become sufficient to induce macroscopic movement of the material. This flow behavior is consistent with that of a Casson fluid, which is characterized by the presence of a yield stress and a nonlinear flow response once flow is initiated [13].

The Casson viscosity η_CA_ and yield stress τ_CA_ values of all hydrogel samples are reported in Table 1. Among them, the P-CO hydrogel exhibited the highest τ_CA_ indicating a greater resistance to flow initiation, likely due to strong intermolecular forces and a more cohesive polymeric network structure. Despite this, the lower Casson viscosity of the P-CO sample, compared to mucilage-based hydrogels, suggests that although the network resists initial deformation, it rapidly breaks down under shear. This implies that the hydrogel undergoes significant structural rearrangements during flow, likely due to the disruption of transient interactions among polymer chains or colloidal systems.

### 2.2. Determination of Dynamic Rheological Properties

**Figure 2:**
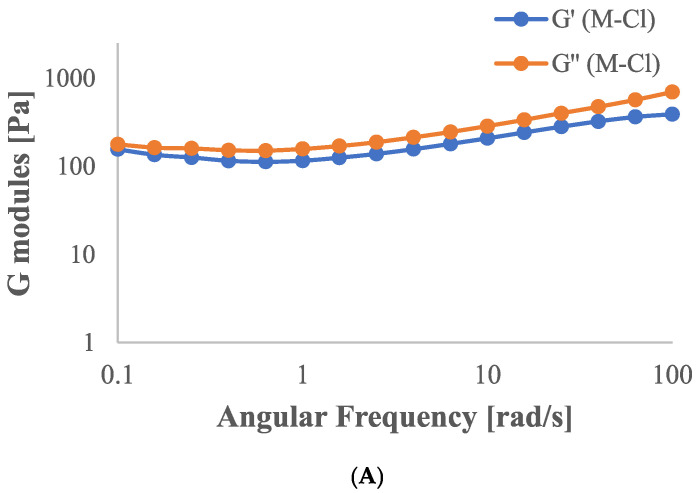

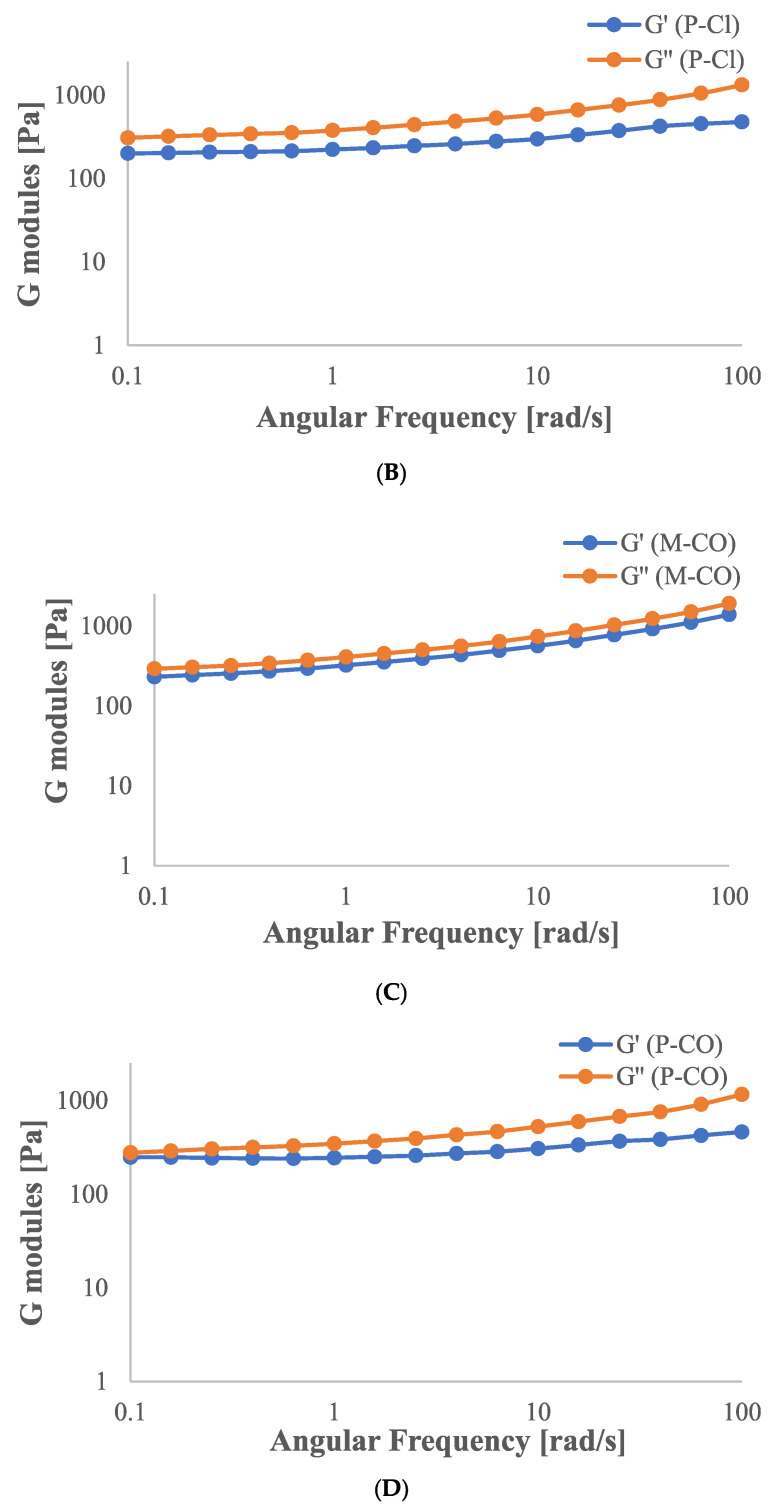
Frequency sweep of (**A**) mucilage added to CaCl_2_ (M-Cl); (**B**) pectin added to CaCl_2_ (P-Cl); (**C**) mucilage added to CaCO_3_ (M-CO); (**D**) pectin added to CaCO_3_ (P-CO).

Frequency sweep rheological measurements (Figure 2) showed that all samples exhibited G″ values consistently exceeding G′ ones throughout the tested frequency range, confirming a predominance of the viscous character over the elastic one.

M-Cl, P-Cl, and P-CO hydrogels (Figure 2A–C)) showed relatively flat G′ curves; in contrast, the presence of calcium carbonate in the mucilage sample (Figure 2C) led to substantial increases in both G′ and G″. Although the sample remained viscous-dominated, the steeper G′ increase indicates enhanced elastic structuring and a more pronounced network character.

To better understand the rheological behavior of the studied hydrogels, the hydrogel viscoelastic properties as a function of angular frequency were analyzed by fitting the storage (G′) and loss (G″) moduli to a power law model, according to Equations (3) and (4).

The obtained exponents and consistency indices, as well as the loss factor (tanδ) evaluated for each hydrogel, are summarized in Table 2.

According to previous results, all samples exhibited tanδ values > 1, indicating viscoelastic systems dominated by the loss modulus. Moreover, looking at the values of tanδ, it is noticeable that the addition of calcium carbonate to both pectin- and mucilage-based hydrogels resulted in a decrease in the damping factor and an increase in k’ and k’’ values, indicating that carbonate made the hydrogel networks stronger [14]. The lowest tanδ value and the highest k′ and k″ values were obtained in the M-CO hydrogel, confirming the presence of a more structured network [15].

The values of n′ and n″ provided information about the dependence of moduli on the frequency. In particular, the investigated hydrogels exhibited frequency-dependent viscoelastic behavior, with n″ > n′ in every case, indicating that the viscous component increases more rapidly with frequency than the elastic one. This trend is generally associated with materials in which energy dissipation dominates over storage at higher deformation rates, a characteristic of weakly structured gel networks [16].

However, the magnitude of the difference between n′ and n″ varied among the samples, providing information on their microstructural features and degree of crosslinking.

Mucilage-based hydrogels showed relatively similar values of n′ and n″, suggesting a more balanced viscoelastic profile. This balance is often interpreted as evidence of a homogeneous network, capable of responding elastically and viscously in a coordinated manner over a broad frequency range [17].

In contrast, pectin-based hydrogels displayed lower n′ and n″ values and larger differences between them, indicative of a structurally weak system, where the viscoelastic behavior is predominantly governed by the viscous, rather than the elastic, response.

In conclusion, the lowest value of tanδ (1.89) observed in the P-Cl hydrogel, coupled with a low n′ and n″ value, suggests a low degree of structuring and a fluid-like behavior. In contrast, the M-CO hydrogel, with the lowest tanδ (1.30) and the highest k′ and k″ values, reflects enhanced elasticity and a more developed viscoelastic network.

### 2.3. ζ-Potential and Particle Size Distribution and Poly Dispersibility Index (PDI)

The resulting data showed that both mucilage and pectin have ζ-potential values significantly lower compared to their relative complex systems (−7.2 mV for mucilage powder dispersed in water and −9.9 mV for pectin powder dispersed in water) (Table 3 and Table 4). In mucilage hydrogels, the value of the ζ-potential became slightly more negative with the addition of the salts (from −9.9 mV related to the single hydrocolloid diluted in water, to −11.0 mV and −14.8 mV for M-Cl and M-CO, respectively) as the pH increased from 4.2 to 5.4, classifying the samples as anionic polyelectrolyte biopolymers.

The ζ-potential is important to evaluate the stability of a hydrogel [18]: if colloids dispersed in a solvent have a low repulsive force, they can interact each other and aggregate. Particles with a ζ-potential between −10 and +10 mV are considered approximately neutral, this means that phase separation will occur, depending on their density compared to the solvent, and the colloidal phase will flocculate or sediment [19], while particles with ζ-potentials more positive than +30 mV or more negative than −30 mV are generally considered stable [19]. Therefore, ζ-potential was analyzed preliminarily in simple hydrocolloids (mucilage and pectin) and then in the complex hydrogels, to evaluate how the presence of calcium salts and sucrose added to the hydrogels would affect the final value of ζ-potential by changing the pH, of which values are reported in Table 5. Pectins were negatively charged at any pH value, due to the presence of carboxyl groups on the surface [20], which dissociated (and released H^+^) as the pH value increased, resulting in higher absolute values of ζ-potential. In pectin systems, the CaCO_3_ could decrease the ζ-potential by −29.1 mV, compared to CaCl_2_. Among the samples analyzed, P-CO showed the highest absolute potential value (−34.5 mV), indicating strong gel stability [21]. The P-Cl sample showed the lowest value of ζ-potentials, which results in faster phase separation of the analyzed colloid.

The pH of pectin systems varied from 2.9 (with CaCl_2_) to 7.8 (with CaCO_3_), changing the ζ-potential values from −5.4 mV to −34.5 mV, respectively. This is an indirect indication of the presence of acidic sugar as units of polysaccharides. In fact, by changing the pH through the use of a different calcium salts, the value of the potential ζ changed significantly in the case of pectin and less in the case of mucilage. This is because pectin can be rich in galacturonic acid units, which are able to dissociate depending on the pH and pKa of the acid. Therefore, Ca^++^ can interact with carboxylate ions and form a stable bridge, as confirmed by the ζ-potential. It was established that the increase in calcium ions could improve the cross-linking density, which indicates a more compact gel structure of the polymers and smaller pore size of the gel network. The mucilage hydrogels showed less significant change in both ζ-potential and pH values than the pectin samples, varying from 4.2 to 5.4 of pH and from −11.5 to −14.8 values (Table 4 and Table 5). A ζ-potential value of about −23 mV for mucilage extracted from the pads of the cactus *Opuntia ficus-indica* was reported by [22].

The relationship between the ζ-potential and viscosity depends on several parameters, such as the particle size distribution (PSD), polydispersity index (PDI), and solid fraction dispersed in the solvent (Table 6). The mucilage samples showed higher Casson viscosity values than the pectin systems, probably because the concentration of suspended solids was higher. The results showed that the M-Cl sample had a higher average particle size (PS = 563.7 d·nm) and a lower polydispersibility index (0.732) than M-Cl (for which the mean PS was 309.8 d·nm and the PDI was 0.99). Systems with such high polydispersity indices are low-viscosity systems, in which smaller particles are able to fit into the gaps between larger particles, allowing the system to flow properly [23]. Considering pectin, the average PSD of the P-CO sample was twice as high (1243 d·nm) as that with calcium chloride (P-Cl: 517.5 d·nm), with a lower dispersibility index (0.427 for P-CO and 0.595 for P-Cl). The particle size of pectin is a crucial factor affecting the viscosity, and pectin with a larger PSD had greater viscosity (0.20 mPa·s for P-CO, higher compared to the P-Cl viscosity of 0.14 mPa·s) [24]. Moreover, the low-polydispersity system indicates the presence of nearly equal-sized particles. Furthermore, the ζ-potential of P-CO was the lowest (−34.5 mV), indicating that the salt was able to form bridges between carboxylate ions of galacturonic acid, forming a stable gel. Lastly, P-Cl samples showed a higher PdI (0.595), indicating a more varied particle size composition and an average PSD about half that of P-CO (517.5 d·nm) and thus a lower viscosity (0.14 mPa·s). These results for the P-Cl sample seem consistent with the instability of the system (ζ-potential: −5.37 mV) and the highest tendency for syneresis (released liquid = 54%).

### 2.4. Released Liquid Mass and Water Activity

None of the hydrocolloids released water after centrifugation, except P-Cl, which instead showed a weight loss of 54 percent. The instability, already shown by the lowest value of ζ-potentials, was confirmed by the released mass analysis in P-Cl samples. Water activity results showed significant differences between pectins and mucilages (Table 7).

### 2.5. Fourier Transform–Infrared Spectroscopic Analysis (FT-IR)

**Figure 3:**
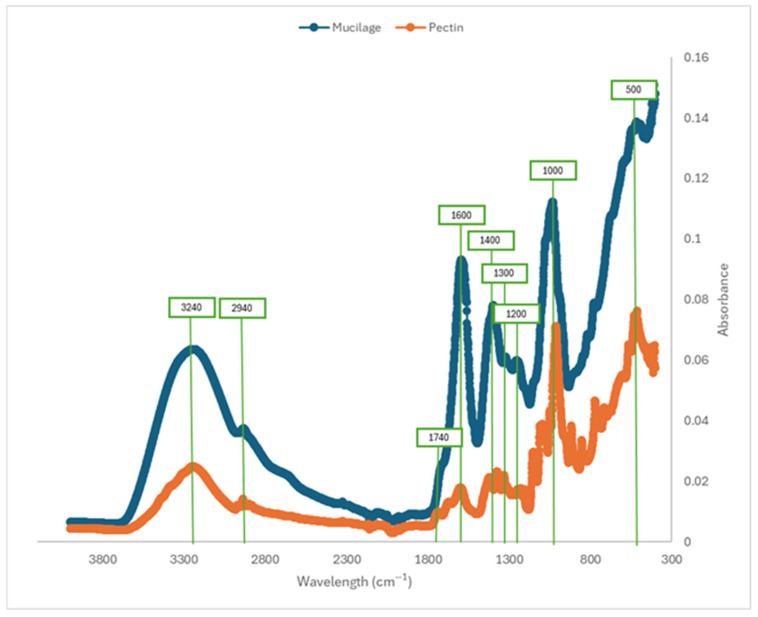
Fourier transform–infrared spectroscopy performed on pectin and mucilage samples.

The region between 800 and 1300 cm^−1^, which can be considered a fingerprint of pectins [25], showed differences between mucilage and pectin samples, especially in relation to the length of their main chain (Figure 3). The band at 3272 cm^−1^, most prominent in mucilages, corresponds to stretches of R-OH groups present in galacturonic acids and sugars, which facilitate the formation of intermolecular hydrogen bonds in the mucilage structure. This interaction is important for the technological properties of mucilage, such as gel formation, film development, and emulsification [26]. The absorption peaks in the range of 1185–1045 cm^−1^ correspond to the stretching vibrations of glycosidic bonds (-CO, ether groups) and pyranose rings (-C-C), which are characteristic of polysaccharide structures. The stretching vibration of the carbonyl group (-C=O) from methyl esterified groups appears in the absorption bands between 1750 and 1735 cm^−1^. Meanwhile, the region between 1650 and 1600 cm^−1^ is associated with carboxyl (-COOH) groups [27]. Additionally, Synytsya et al. [26] reported that the stretching vibrations of carboxylate anions (-COO^−^) are observed at 1633 cm^−1^, while the absorption band at 1320 cm^−1^ corresponds to the methyl (-CH) vibration of the rhamnose ring, a common component of mucilage derived from Opuntia species. The functional groups identified via IR spectroscopy can be used to infer chemical interactions influenced by functional groups such as hydroxyl, carboxyl, and methyl, suggesting potential binding, stabilization, and water-retention properties that enhance their utility as functional ingredients in food products.

### 2.6. Multivariate Evaluation of Gels Physical-Chemical Properties

**Figure 4:**
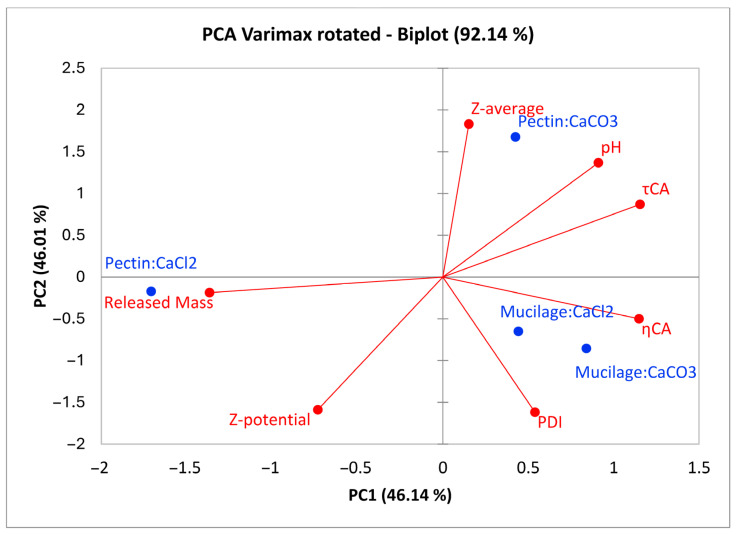
Biplot of principal component analysis with Varimax rotation of the prepared gels; the correlation values of variables with PC1 are multiplied by 1.386 and 1.883 for PC2 as graphical rescaling.

Principal component analysis (PCA) performed with Varimax rotation explained 92.14% of the total variance across the first two principal components. PC1 was highly positively correlated (Pearson’s R ≥ 0.7) with τ_CA_ and η_CA_ and highly negatively correlated (Pearson’s R ≤ −0.7) with the released mass. PC2 was highly positively correlated with the pH and Z-average and highly negatively correlated with the Z-potential and PDI. The results show that pectin gels respond very differently depending on the calcium salt used during preparation. CaCO_3_ induces greater aggregate formation compared to CaCl_2_, as evidenced in Figure 4, where P-CO is associated with a higher Z-average, a higher pH, and increased τ_CA_ and η_CA_ values. These findings suggest a higher content of dissociable acidic sugars, for which carboxylate groups can interact with calcium ions to form larger aggregates than those formed with CaCl_2_. This interaction also results in higher viscosity and greater stability, as indicated by a lower ζ-potential compared to the gel prepared with pectin and CaCl_2_. Moreover, the use of calcium carbonate leads to a more stable dispersion, as suggested by the relative positions of released mass and P-CO in Figure 4. Mucilages showed a similar multivariate profile (based on the measured variables), generally displaying a greater polydispersity compared to pectic gels, along with higher τ_CA_ and η_CA_ values. Additionally, the chemical–physical properties of mucilage gels appeared to be less influenced by the type of calcium salt used (Figure 4).

## 3. Conclusions

This study provides a comparison of the structural and rheological characteristics of mucilage extracted from *Opuntia ficus* cladodes with commercial pectin. All samples showed non-Newtonian behavior and the results obtained from the sweep test showed a good fit to the power law equation, confirming the viscoelastic behavior of both hydrogels. Resulting data showed that both mucilage and pectin powders had ζ-potential values significantly lower compared to their relative complex systems. The IR analysis confirmed it can be the fingerprint of all polysaccharides, showing clear differences between mucilage and pectin samples. In addition, the functional groups identified via IR spectroscopy could be used to infer chemical interactions between different chemical groups and some functional properties of mucilage as ingredients in food products. Overall, the reported results highlighted the potential applications of mucilage solutions from Opuntia cladodes as gelling agents in industrial food processes.

## 4. Materials and Methods

### 4.1. Extraction and Freeze-Drying of Cactus Pear Mucilage

One-year-old cladodes were collected from 10-year-old *O. ficus-indica* plants, cv. *Gialla*, spaced 6 × 5 m apart and trained to a globe shape. The commercial orchard was located in Roccapalumba, Palermo, Italy (37°48′ N, 13°38′ E, 350 m a.s.l) on sandy-loam Mediterranean red-soils, and plants were subjected to ordinary horticultural care. Harvested cladodes were labeled, packaged, and moved to the laboratory, where they were weighed and processed for mucilage extraction using a modified patented method by Du Toit and De Witt [28]. To increase the shelf life of the mucilage and get rid of contaminants and spines, cladodes were cleaned with chlorinated water (200 mg kg^−1^ of sodium hypochlorite). The cladode chlorenchyma was removed with a peeler to obtain high-quality mucilage from the parenchyma; cladodes were then sliced into squares and cooked in a microwave oven (900 W) for 3–5 min until soft. An Omni Mixer Homogenizer (mod. Omni-Mixer. 17107, Dupont Instruments Sorvall, Modesto, CA, USA) was then used to mix the cooked, soft cladode pieces to aid in the mucilage extraction. The obtained pulp was then centrifuged using a Sigma centrifuge (mod. 6K15, Sigma Laborzentrifugen GmbH, Osterode am Harz, Germany) at 8000× *g* for 15 min at 4 °C. The mucilage yield extracted from the cladodes was about 20%. After that, the supernatant mucilage was collected in a plastic container and stored in a freezer at −20 °C until lyophilization occurred. The freeze drying was performed with a SCANVAC Coolsafe 55-9 (DK-3450, Labogene ApS Lynge, Lynge, Denmark). After freeze-drying, mucilage lost about 98% of its moisture content; but for large-scale applications, further studies will be needed to evaluate more sustainable drying systems. The mucilage was then stored in a freezer at −27 °C until its use for preparing hydrogel samples and the succeeding analysis.

### 4.2. Mucilage and Pectin Hydrogels Preparation

High methoxyl pectins (HMPs) were provided by Distillerie Ruffini S.r.l., Sambuca (FI), Italy. HMPs have a large part of galacturonic acid esterified with methanol [29], needing at least 40% of sucrose in solution to start forming a gel. Calcium chloride and carbonate calcium (Sigma Aldrich, St. Louis, MO, USA) (1% *W*/*V*) were added to let the divalent ions (Ca^2+^) interact with the remaining carboxylate ions of galacturonic acid, so as to achieve better gel network formation. To have a similar composition in the different systems, sucrose was also added to the mucilage systems at the same concentration. Considering that pectin is mainly composed of D-galacturonic acid monomers [30] and that in the mucilage of OFI cladodes, the concentration of D-galacturonic acid is about 23% (without distinction between methyl ester form and acidic form) [31], the concentrations used for pectin and mucilage in the hydrogels were 4% and 20%, respectively. These different concentrations may seem large, but can be functional, especially if the primary goal is to enhance the viscosity or texture. Ingredients were mixed and placed in a glass baker in a 0.1 M citrate buffer at pH 3.5, with continuous magnetic stirring until samples reached a 100 °C temperature, kept under control by a probe thermometer. Bakers were sealed with aluminum foil to avoid water loss during boiling. All analyses were conducted on the hydrogels and, only in specific cases, also on the powder of hydrocolloids diluted in water. In particular, the ζ-potential was measured on both hydrocolloids (freeze-dried mucilage and pectin) diluted in water and on the prepared hydrogels to evaluate the surface charge of mucilage and pectin particles, with and without the influence of other components (sucrose and calcium salts) in the gels. The composition and the code of the pectin and mucilage gels is reported in Table 8.

### 4.3. Rheological Measurements

The rheological characterization of hydrogel samples was performed with a rotational rheometer (Modular Compact Rheometer MCR 102e, Anton Paar, Graz, Austria) with plate–plate geometry and a final gap of 1 mm at a constant temperature (25 °C) controlled by a Peltier system in combination with an external thermo-cryostat (Argo Lab CB 5-30, Geass, Torino, Italy). In all the experiments, the samples were surrounded by silicone oil to prevent moisture loss. All measurements were conducted in triplicate.

#### 4.3.1. Flow Curve Analysis

Pectin and mucilage hydrogels were characterized regarding their steady-shear viscosity, measuring it as function of the increasing shear rate ranging from 0.1 and 100 s^−1^. To assess the rheological behavior of the samples, Casson’s model (Equation (1)) was found to be the best fitting equation.

(1) $$\sqrt{\tau }=\sqrt{\tau_{CA}}+\sqrt{\eta_{CA}}\sqrt{\dot{\gamma }}$$

where τ is the shear stress (Pa), $\tau_{CA}$ is Casson’s yield stress (Pa), $\eta_{CA}$ is Casson’s plastic viscosity (Pa s), and $\dot{\gamma }$ is the shear rate (s^−1^).

By observing the linearized Casson rheological model, it immediately becomes clear that the slope of the model, that is, the variation of τ with respect to the variation of $\dot{\gamma }$, is not constant.

(2) $$\sqrt{\tau }=\sqrt{\tau_{CA}}+\sqrt{\eta_{CA}}\sqrt{\dot{\gamma }}$$

Deriving τ,

(3) $$\tau ={\left(\sqrt{\tau_{CA}}+\sqrt{\eta_{CA}\dot{\gamma }}\right)}^{2}$$

Expanding the binomial square,

(4) $$\tau =\tau_{CA}+2\sqrt{\tau_{CA}\eta_{CA}\dot{\gamma }}+\eta_{CA}\dot{\gamma }$$

By determining the derivative, $\frac{d\tau }{d\dot{\gamma }}$

(5) $$\frac{d\tau }{d\dot{\gamma }}=\eta_{CA}+\sqrt{\frac{\tau_{CA}\eta_{CA}}{\dot{\gamma }}}$$

Consequently, evaluating only the parameter η_CA_ is not sufficient to describe the variation in the shear stress as a function of the variation in the shear rate, and then, all the coefficients of Casson’s model and the shear rate must be considered.

#### 4.3.2. Viscoelastic Behavior

The viscoelastic properties of each hydrogel, in terms of the storage modulus (G′, Pa), loss modulus (G″, Pa), and tan *δ* (G″/G′) were measured as function of the angular frequency (ω, rad/s), in the range of 0.1 to 100 rad/s, with shear strain at 1%. Before measuring the viscoelastic properties, a strain sweep pre-test was carried out to define the linear viscoelasticity zone, where the shear stress varies linearly with the applied strain. The pre-test at a constant frequency of 1 Hz allowed the upper limit of the linear viscoelastic zone to be fixed at a strain of 1%.

The frequency sweep output was analyzed using a Heschel–Bulkley model applied to the sweep test, to define the dependence of the storage and loss moduli, G′ and G″, based on the angular frequency (*ω*), according to Equations (6) and (7) [32]:

(6) $$G^{\prime }=k\prime {\left(\omega \right)}^{n\prime }$$

(7) $$G^{\prime \prime }=k\prime \prime {\left(\omega \right)}^{n\prime \prime }$$

where k′ and k″ are the consistency indices for G′ and G″, respectively, and n′ and n″ are the frequency-dependence exponents.

### 4.4. ζ-Potential, Particle Size Distribution, and Polydispersity Index

The particle size distribution, ζ-potential, and polydispersity index were analyzed with a Dynamic Light Scattering, Malvern Zetasizer Nano ZS (Malvern Instruments Ltd., Malvern, Worcestershire, UK). The particle size distribution was evaluated for complete hydrogel systems diluted in ultra-pure water at 1:10 for pectin hydrogels and at 1:100 for mucilage hydrogels. The analysis was conducted with samples placed in a disposable sizing cuvette, at a constant temperature of 25 °C for 50 s of measurement.

The ζ-potential was analyzed for complete hydrogel systems and for freeze-dried powders separately, pectin and mucilage, without any other component. Samples were placed with a syringe in a DTS1070 cell (Malvern Panalytical, Malvern, UK), at a constant temperature of 25 °C. All measurement were performed in triplicate.

### 4.5. Released Liquid Mass (RM), Water Activity (aW), and pH

The capability of all hydrogel systems to retain water was measured according to Ciron et al. [33] spinning at 1350 rpm for 60 min, in the centrifuge ALC 4237, and the water shaken off was absorbed by filter papers. The RM was calculated as follows (Equation (8)):

(8) $$RM(\%)=\frac{w_{R}}{w_{T}}100$$

where w_R_ is the liquid mass separated after the gel centrifugation. W_T_ is the mass of the gel before the centrifugation.

The water activity was evaluated throughout the use of the HygroPalm-23 instrument (Rotronic, Basserdorf, Germany) according to the ISO 18787:2017 method [34]. The analysis was conducted at a constant temperature of 21 °C ± 0.5, in triplicate. A GLP21 pH-meter (CRISON 08328, Barcelona, Spain) was used to measure the pH of the samples.

### 4.6. Fourier Transform–Infrared Spectroscopic Analysis (FT-IR)

The study of functional groups was carried out using a Fourier transform–infrared (FT-IR) spectrometer (Perkin Elmer FTIR UATR, Waltham, MA, USA). Mucilage and pectin samples (10 mg) were placed on the specular reflectance plate. The samples were analyzed in the 4000–600 cm^−1^ region, at a resolution of 4 cm^−1^ (20 scans).

### 4.7. Statistical Analysis

A two-way ANOVA (hydrocolloid and calcium salt as factors) with interaction analysis was used to test the influence of the gelling agent (pectin or mucilage), calcium salts, (calcium chloride or carbonate calcium), and the interaction between them on the water activity, Casson viscosity, pH, ζ-potential, and color measurements. The results shown in the tables explain how the variable considered (hydrocolloid or salt or their interaction) had statistical influence on the result of the measurement.

One-way analysis of variance ANOVA was conducted with R 4.1.3 (R Foundation for Statistical Computing, Vienna, Austria) for all measurements. Means and standard deviations reported for the factorial model were calculated as the average and standard deviation of the factors (hydrocolloid and salt) and of the interaction (hydrocolloid:salt).

### 4.8. Multivariate Evaluation of Gel Physical–Chemical Properties

Principal component analysis with varimax rotation on mucilage samples was performed on Z-standardized data using XLSTAT 2023 25.3 trial version (Lumivero, Denver, CO, USA), and the variables used for the analysis were as follows: Z-average, pH, τ_CA_, η_CA_, polydispersion index, Z-potential, and released mass.

## Figures and Tables

**Table 1:** Casson’s viscosity values and Casson yield stress of hydrogels.

Sample	Casson Viscosity *η_Ca_* [mPa·s]	Casson’s Yield Stress *τ_Ca_* [Pa]
M-Cl	64.26 ± 5.2	4.01 ± 1.0
M-CO	52.62 ± 4.8	5.50 ± 0.8
P-Cl	24.40 ± 3.1	3.44 ± 1.0
P-CO	30.87 ± 2.9	11.48 ± 2.0

**Table 2:** Slopes (n′, n″) and intercepts (k′, k″) of ln (G′, G″) and tanδ values evaluated for hydrogel samples.

Hydrogels	k′	k″	n′	n″	tanδ
M-Cl	154.88	109.65	0.28	0.34	1.38
M-CO	371.53	295.12	0.31	0.33	1.30
P-Cl	346.74	208.93	0.18	0.26	1.89
P-CO	316.23	229.09	0.14	0.25	1.61

**Table 3:** Values of ζ-potential for hydrogel samples: pectin added to CaCl_2_ (P-Cl); pectin added to CaCO_3_ (P-CO); mucilage added to CaCl_2_ (M-Cl); mucilage added to CaCO_3_ (M-CO). Multifactorial analysis performed on values of ζ-potential for hydrocolloids, salts, and their interaction (hydrogel samples) as a final result.

Sample	ζ-Potential [mV]
Hydrocolloid	
Mucilage	−13 ± 2
Pectin	−20 ± 16
Sign.	***
Salt	
CaCl_2_	−8 ± 3
CaCO_3_	−25 ± 11
Sign.	***
Hydrogel samples	
M-Cl	−11.5 ± 0.6 ^b^
M-CO	−14.8 ± 0.2 ^c^
P-Cl	−5.4 ± 0.5 ^a^
P-CO	−34.5 ± 0.5 ^d^
Sign.	***

Data expressed as the mean ± standard deviation. *** = 99.9% ANOVA significance. Different letters (a, b, c, d) in the same row mean statistical difference (*p* < 0.05) among samples. Anova F test significativity: *** = *p* value < 0.001.

**Table 4:** Values of ζ-potential measured for hydrocolloids diluted in water. The Δζ-potential is the difference between values of ζ-potential obtained using hydrogel samples and that obtained using the relative hydrocolloid only diluted in water.

**Hydrocolloid in water**	**ζ-Potential [mV]**
Mucilage	−7.2 ± 0.5
Pectin	−9.9 ± 0.5
Sign.	**
**Hydrogel Samples**	**Δζ-potential [mV]**
M-Cl	−4.3 ± 0.09
M-CO	−7.6 ± 0.68
Sign.	**
P-Cl	4.6 ± 0.9
P-CO	−24.6 ± 0.4
Sign.	***

Data expressed as the mean ± standard deviation. *** = 99.9% ANOVA significance. Anova F test significativity: ** = 0.001 ≤ *p* value < 0. 01, *** = *p* value < 0.001.

**Table 5:** Values of pH expressed as mean and standard deviation, for hydrogel samples: pectin added to CaCl_2_ (P-Cl); pectin added to CaCO_3_ (P-CO); mucilage added to CaCl_2_ (M-Cl); mucilage added to CaCO_3_ (M-CO). Multifactorial analysis performed for hydrocolloids, salts, and their interaction (hydrogel samples) as a final result.

Factor	pH
Hydrocolloid	
Mucilage	4.8 ± 0.7
Pectin	5.4 ± 2.6
Sign.	***
Salt	
CaCl_2_	3.6 ± 0.7
CaCO_3_	6.6 ± 1.3
Sign.	***
Hydrogel Samples	
M-Cl	4.22 ± 0.02 ^c^
M-CO	5.4 ± 0.3 ^b^
P-Cl	2.95 ± 0.03 ^d^
P-CO	7.77 ± 0.01 ^a^
Sign.	***

Data expressed as the mean ± standard deviation. *** = 99.9% ANOVA significance. Different letters (a, b, c, d) in the same row mean statistical difference (*p* < 0.05) among samples. Anova F test significativity: *** = *p* value < 0.001.

**Table 6:** Values of particle size distribution (Z-average), polydispersibility index (PDI), Casson viscosity, and Z-potential for pectin added to CaCl_2_ (P-Cl), pectin added to CaCO_3_ (P-CO), mucilage added to CaCl_2_ (M-Cl), and mucilage added to CaCO_3_ (M-CO) expressed as the mean ± standard deviation.

Sample	Z-Average (d·nm)	PDI	Casson Viscosity [mPa·s]	Z-Potential
P-Cl	517.5	0.595	65 ± 16	−5.37 ± 0.53
P-CO	1243	0.427	49 ± 21	−34.50 ± 0.50
M-Cl	563.7	0.732	20 ± 11	−11.50 ± 0.56
M-CO	309.8	0.99	42 ± 12	−14.83 ± 0.21

**Table 7:** Water activity measured for pectin added to CaCl_2_ (P-Cl), pectin added to CaCO_3_ (P-CO), mucilage added to CaCl_2_ (M-Cl), and mucilage added to CaCO_3_ (M-CO) with multifactorial analysis evaluated for hydrocolloids, salts, and their interaction (hydrogel samples) as a final result. Released liquid mass measured after applied centrifugation based on hydrogel samples: pectin added to CaCl_2_ (P-Cl); pectin added to CaCO_3_ (P-CO); mucilage added to CaCl_2_ (M-Cl); mucilage added to CaCO_3_ (M-CO).

Variable/Factor	aW	
Hydrocolloid		
Mucilage	0.93 ± 0.02	
Pectin	0.95 ± 0.01	
Sign.	*	
Salt		
CaCl_2_	0.94 ± 0.02	
CaCO_3_	0.94 ± 0.02	
Sign.	ns	
Hydrogel Samples	aW	Released Mass [% *w*/*w*]
P-Cl	0.95 ± 0.01	<LOQ ^b^
P-CO	0.95 ± 0.02	<LOQ ^b^
M-Cl	0.92 ± 0.02	54 ± 3 ^a^
M-CO	0.93 ± 0.01	<LOQ ^b^
Sign.	ns	***

Data expressed as the mean ± standard deviation. *** = 99.9% ANOVA significance. Different letters (a, b, c, d) in the same row mean statistical difference (*p* < 0.05) among samples. Anova F test significativity: ns = no significant difference, * = 0.01 ≤ *p* value < 0.05, *** = *p* value < 0.001.

**Table 8:** Sample formulation for hydrogels with pectin (P-Cl and P-CO) and mucilage (M-Cl and M-CO).

Sample Coding	Water	Sucrose	Gelling Agent	Calcium Salt (1%)
(P-Cl)	25 mL	10 g	1.0 g pectin	0.25 g CaCl_2_
(P-CO)	25 mL	10 g	1.0 g pectin	0.25 g CaCO_3_
(M-Cl)	25 mL	10 g	5.0 g mucilage	0.25 g CaCl_2_
(M-CO)	25 mL	10 g	5.0 g mucilage	0.25 g CaCO_3_

## Data Availability

The original contributions presented in this study are included in the article. Further inquiries can be directed to the corresponding author.
